# GM-lncLoc: LncRNAs subcellular localization prediction based on graph neural network with meta-learning

**DOI:** 10.1186/s12864-022-09034-1

**Published:** 2023-01-28

**Authors:** Junzhe Cai, Ting Wang, Xi Deng, Lin Tang, Lin Liu

**Affiliations:** 1 School of Information, Yunnan Normal University, Kunming, Yunnan China; 2 Key Laboratory of Educational Information for Nationalities Ministry of Education, Yunnan Normal University, Kunming, Yunnan China

**Keywords:** lncRNA, Subcellular localization, Graph neural network, Meta-learning, Classification

## Abstract

In recent years, a large number of studies have shown that the subcellular localization of long non-coding RNAs (lncRNAs) can bring crucial information to the recognition of lncRNAs function. Therefore, it is of great significance to establish a computational method to accurately predict the subcellular localization of lncRNA. Previous prediction models are based on low-level sequences information and are troubled by the few samples problem. In this study, we propose a new prediction model, GM-lncLoc, which is based on the initial information extracted from the lncRNA sequence, and also combines the graph structure information to extract high level features of lncRNA. In addition, the training mode of meta-learning is introduced to obtain meta-parameters by training a series of tasks. With the meta-parameters, the final parameters of other similar tasks can be learned quickly, so as to solve the problem of few samples in lncRNA subcellular localization. Compared with the previous methods, GM-lncLoc achieved the best results with an accuracy of 93.4 and 94.2% in the benchmark datasets of 5 and 4 subcellular compartments, respectively. Furthermore, the prediction performance of GM-lncLoc was also better on the independent dataset. It shows the effectiveness and great potential of our proposed method for lncRNA subcellular localization prediction. The datasets and source code are freely available at https://github.com/JunzheCai/GM-lncLoc.

## Introduction

The RNAs that cannot encode proteins are called non-coding RNA (ncRNA) [1], which can be further divided into two categories according to their molecular chain length: small non-coding RNA (sncRNA) with molecular chain length less than 200 nucleotides and long non-coding RNA (lncRNA) with molecular chain length more than 200 nucleotides [2]**.** In the past, lncRNAs were initially considered as the “noise” of genome transcription, which was the by-product of RNA polymerase II transcription and had no biological function [3]**.** However, more and more studies have shown that lncRNAs are involved in many biological functions. Moreover, abnormal behavior of lncRNAs leads to the formation of several types of cancer, Alzheimers disease, Huntingtons disease, and cardiovascular diseases [4–13]**.** Obviously, a better understanding of lncRNA function would enhance our understanding of specific cell development and physiology. Several studies have shown that the function of lncRNA is highly dependent on its position inside the cell [14–16]**.** Therefore, identification of lncRNA subcellular localization is particularly important.

There are two main types of methods for predicting lncRNA subcellular localization. One is biochemical experiments, which have the advantage of precise positioning results and have the disadvantage of being time-consuming and expensive. Therefore, more and more researchers have tried to find a breakthrough in computational methods, which have the advantages of being time-saving, efficient and stable. Especially with the solid foundation provided by lncRNA subcellular localization databases, including RNALocate [17], LncATLAS [18], and lncSLdb [19], computational model-based lncRNA subcellular localization methods have become a new trend in this field of research.

At present, several computational models have been used to predict the subcellular localization of proteins with high accuracy [20–24]**.** Such problem of protein or RNA subcellular localization prediction is essentially a classification process in machine learning. Therefore, the current studies also follow the general process of classification prediction, including dataset building, lncRNA feature extraction, and classifier training. Zhen C, et al. [25] proposed the lncLocator method, which utilizes support vector machines (SVM), Random Forest (RF) and neural network (NN) to predict subcellular localizations of lncRNAs and yield an overall accuracy of 59.1% on benchmark dataset with 5 subcellular compartments; Gudenas, B.L., et al. [26] and Yang Lin, et al. [27]**.** developed the deep learning algorithm to predict subcellular location on a large dataset with 2 classes; Furthermore, several researchers focus on the benchmark dataset with 4 subcellular compartments. Generally, SVM algorithm is widely used as the classification model in predicting lncRNA subcellular localization, such as iLoc-lncRNA proposed by Su Z D, et al. [14], Locate-R proposed by Aa A, et al. [28] and Xiao-Fei Yang, et al. [29], which get an accuracy of 86.11, 90.69 and 92.38%, respectively; also for the benchmark dataset with 4 subcellular compartments, Fan Y, et al. [30] come up with a method based on logistic regression, lncLocPred, which obtains 92.37% accuracy.

Although these aforementioned methods have made some progress in lncRNA subcellular localization prediction, the prediction accuracy varies greatly due to the different label and sample numbers of datasets. Gudenas, B.L., et al. [26] and Yang Lin, et al. [27] utilize a large amount of data and less subcellular localization labels, so relatively high prediction accuracy is obtained. In the rest of studies [14, 25, 28–30], the dataset contains only a few hundred samples with 4 or 5 subcellular compartments, which belongs to the few-shot learning field. From the perspective of computational models, a small number of samples is a big obstacle to the training of classifier, which significantly limits the improvement of prediction accuracy. Especially for the deep learning methods, it is able to automatically capture advanced features of data, but it is not good at getting better generalization performance for few-shot learning. Therefore, for the dataset with 4 or 5 subcellular compartments, previous studies mainly made use of traditional machine learning methods to predict lncRNA subcellular localization and spent a lot of resources on feature extraction. For instance, Zhen C, et al. [25] specially used an unsupervised stacked autoencoder model to obtain high-level features from k-mer low-level features; Fan Y, et al. [30] utilized k-mer, PseDNC and TRIPLET methods to extract features, and then fused these features through a series of operations. Although some recent studies have tried to utilize deep learning to predict lncRNA subcellular localization on dataset with a few lncRNAs, they have obtained poor performance. As an example, the accuracy of DeepLncLoc proposed by Zeng M, et al. [31] is only 53.7% in the dataset with 5 subcellular compartments.

In view of above problems, this paper proposed a new prediction model called GM-lncLoc, which mainly explores how to predict lncRNA subcellular localization in a few samples dataset based on advanced lncRNA features automatically extracted by deep learning. On the one hand, GNN [32] is a powerful model that can aggregate the node features and the information of graph structure, which is conducive to the node classification task of lncRNA subcellular localization research. Therefore, after extracting the low-level features of lncRNA sequences by simple k-mer method, the hidden representation of lncRNA sequences is automatically captured as high-level features based on GNN in our model. On the other hand, meta-learning is an efficient way for dealing with few-shot learning that extracts meta-knowledge from multiple similar tasks, allowing the predictor to acquire the ability of other similar classification tasks quickly. In the field of meta-learning, there are many models that are widely accepted and considered effective, such as MAML [33] and Reptile [34]**.** Inspired by the study of Kexin Huang [35] et al., we attempted to combine GCN [36] and MAML [33] to address the poor performance of deep learning in few-shot lncRNA subcellular localization learning. Generally speaking, GM-lncLoc not only obtains efficient lncRNA subcellular localization prediction on a small number of lncRNA samples, but also learns the meta-parameters with strong generalization ability for rapid adaptation to similar unseen task.

**Fig. 1:**
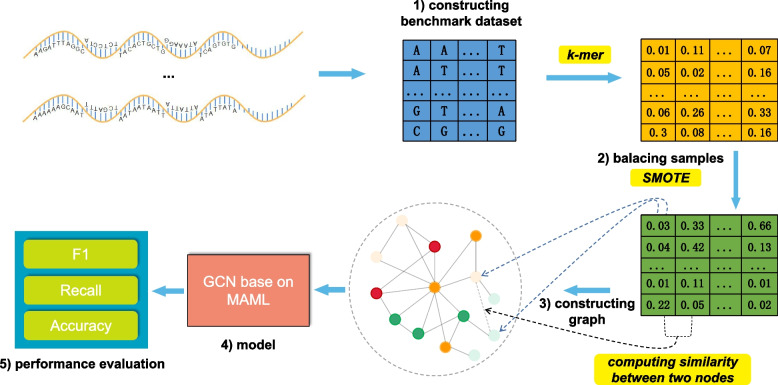
The flow chart of GM-lncLoc: (1) constructing benchmark dataset; (2) balancing samples; (3) constructing graph; (4) predicting labels with the model (GCN based on MAML); (5) evaluating the model’s performance with evaluation indicators

To our best knowledge, we are the first to identify lncRNA subcellular localization based on GNN and few-shot learning method. In general, the steps of GM-lncLoc are as follows: (1) constructing benchmark dataset; (2) balancing samples; (3) constructing graph; (4) Model: GCN based on MAML; (5) performance evaluation. See the flow chart in Fig. 1.

## Materials and methods

### Dataset

**Fig. 2:**
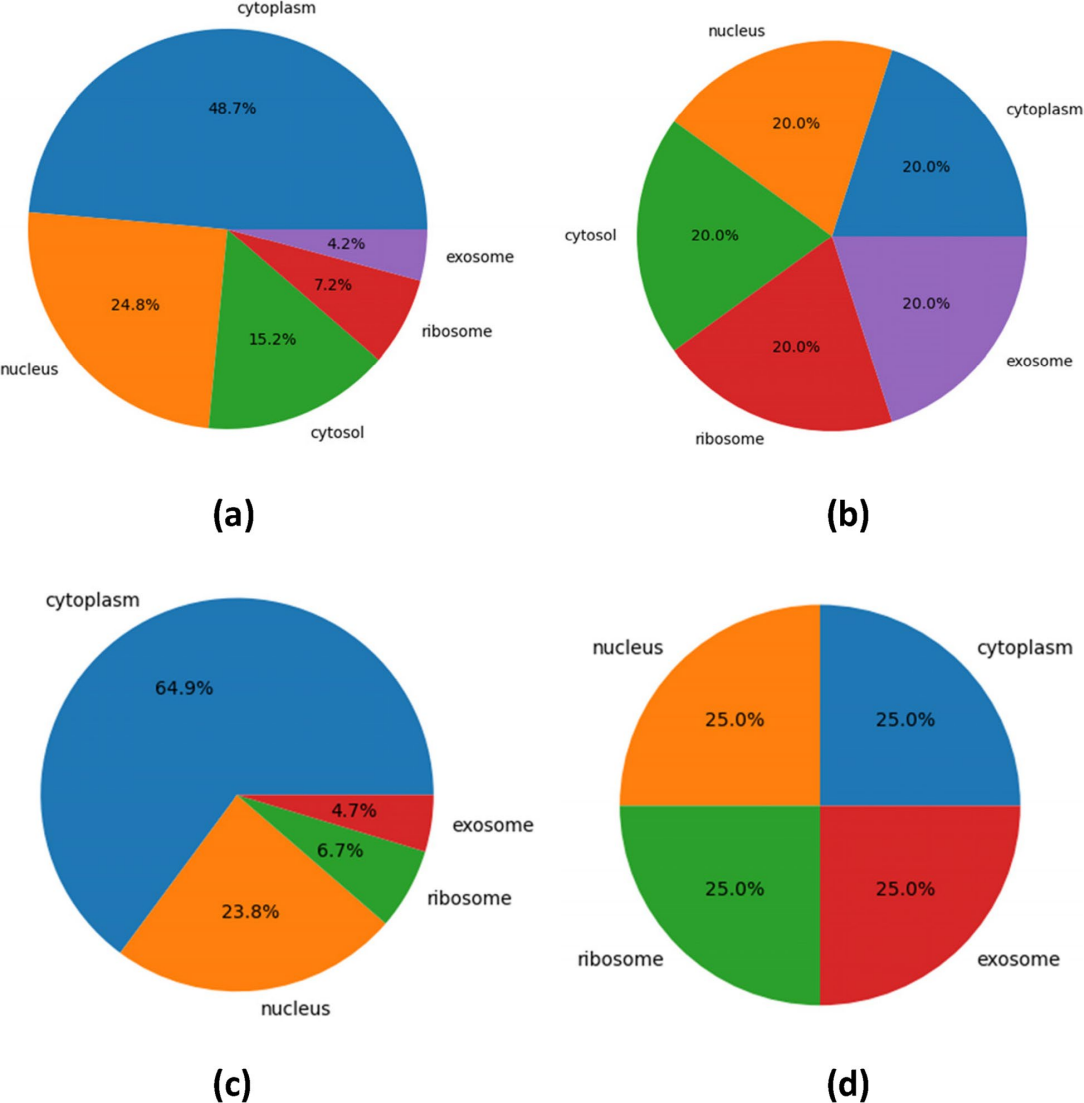
(**a**): dataset1 before SMOTE; (**b**): dataset1 after SMOTE; (**c**): dataset2 before SMOTE; (**d**): dataset2 after SMOTE

A high-quality dataset is crucial for effective and accurate prediction models, where the labels in the dataset are evenly distributed and have sufficient samples. As mentioned above, in the current studies of lncRNA subcellular localization prediction, researchers have mainly constructed three benchmark datasets: Zhen C, et al. [25] and Zeng M, et al. [31] constructed the 5 subcellular compartments dataset from RNALocate database; Gudenas, B.L., et al. [26] and Yang Lin, et.al [27]**.** constructed datasets with 2 subcellular compartments; other researchers have constructed datasets with 4 subcellular compartments. This section mainly introduces the construction of our two datasets, dataset1 and dataset2, which are based on the 5 subcellular compartments dataset of Zhen C, et al. [25] and the 4 subcellular compartments dataset of Su Z D, et al. [14], respectively. The steps of dataset construction are as follows:

- Step 1: First, we download the raw data of Zhen C, et al. [25] and Su Z D, et al. [14] from the websites,^1^ which contain 612 and 655 lncRNAs sequence, as shown in Table 1. After screening, to reduce information redundancy and noise interference, we removed 1 sequence of length 91,671 and 11 sequences containing special symbols “N, R, S and Y”. Finally, dataset1 and dataset2 contain 600 and 643 lncRNAs sequences, respectively, including 292/417 Cytoplasm, 149/153 Nucleus, 91/− Cytosol, 43/43 Ribosome, 25/30 Exosome.
- Step 2: Previous studies have shown that there are many factors related to lncRNA subcellular localization, such as sequence and structure [37]**.** As it is still challenging to identify RNA structural information experimentally and theoretically [38], the approaches of current studies mainly extracted low-level features from lncRNA sequence [14, 25–31] based on k-mer [39], RevKmer [40, 41] and PseDNC [42–44] et al. K-mer can get the basic information of a sequence, and has a wide range of applications in many fields of bioinformatics [45–48]**.** In our experiment, the features of RNA sequences extracted by k-mer have been verified to be more effective than other feature extraction methods. Therefore, after extracting the low-level features of 600/643 lncRNAs sequences by k-mer, 600/643 vectors were obtained.
- Step 3: As shown in Fig. 2**(a)(c)**, the dataset is unbalanced with a few samples. At present, there are two main methods to balance samples: under-sampling and over-sampling. The under-sampling method randomly selects the subsets of samples from each classification to consist of a balanced dataset [49, 50], which will led to loss of important information from original data.

**Table 1:** Benchmark dataset

	Original1	After filtering	After SMOTE (dataset1)	Original2	After filtering	After SMOTE (dataset2)	dataset3
Cytoplasm	301	292	292	426	417	417	198
Nucleus	152	149	292	156	153	417	82
Cytosol	91	91	292	–	–	–	–
Ribosome	43	43	292	43	43	417	99
Exosome	25	25	292	30	30	417	16
Total	612	600	1460	655	643	1668	395

However, the over-sampling method synthesizes new data for labels with only a few samples, which is more suitable for small and unbalanced dataset and it is also adopted in many other studies, such as lncLocator [25], Locate-R [28], and so on. Therefore, an over-sampling method Synthetic Minority Over-Sampling Technique (SMOTE) [51] is considered in this paper. Taking dataset1 as an example, SMOTE synthesizes the data as follows: (1) 292, the number of Cytoplasm classes with the largest number of samples in the original dataset, was chosen as a reference; (2) randomly select a sample in the Nucleus class as the central sample, and 143 nearest neighbors of this center sample were selected stochastically; (3) 143 samples are randomly generated along the line segments of the central sample and 143 nearest neighbors, and then the Nucleus class contains 292 samples, including 149 real and 143 synthetic samples; (4) the samples of Cytosol, Ribosome and Exosome were sampled according to (2) and (3), and finally, 292 samples were collected for each class. There are 1460/1668 samples in total in the final datasets after over-sampling. It can be seen in Table 1 and Fig. 2**(b)(d)** that the distribution of labels in final datasets is balanced. However, the sample size is not enough to support the deep learning model to get good results.

Moreover, we prepare an independent test set, dataset3, provided by Fan Y, et al. [30]**.**^2^ We removed 1 sequence containing special symbols and got 395 samples, including 198 Cytoplasm, 82 Nucleus, 99 Ribosome and 16 Exosome.

### Constructing graph

Constructing graph is a process of modifying the data format of low-level features into graphical data, which can be applied to GCN with the advantage of capturing structural information of the graph. In the field of bioinformatics, several researchers have constructed a protein sequence similarity network (SSN) [52–54] to study the properties of proteins. Correspondingly, the graph structure is constructed by cosine similarity of features in this paper. Meanwhile, GM-lncLoc is able to extract information from the perspective of non-Euclidean space, which is the most different from previous methods based on Euclidean space data. An appropriate graph structure facilitates GCNs to aggregate neighbor node information more efficiently.

#### Problem Formulation

The graph is denoted as ***G =*** (***V***, ***E***, ***X***), where ***V =*** {***v***_**1**_, ***v***_**2**_, …, ***v***_***n***_} represents the node-set, ***v***_***i***_ represents the i-th lncRNA sequence, which is one of the nodes in the graph ***G***. ***E =*** {***e***_**1,2**_, ***e***_**1,3**_, …, ***e***_***i,j***_} represents edge-set, ***e***_***i,j***_ represents the edge constructed between the i-th and j-th lncRNA sequence, ***e***_***i,j***_ ***=*** **1** represents the existence edge, and ***e***_***i,j***_ ***=*** **0** represents the non-existence edge. ***X =*** {***x***_**1**_, ***x***_**2**_, …, ***x***_***n***_} represents the node features and ***x***_***i***_ is the initial feature vector of the node ***v***_***i***_ ***∈ V*** in the graph ***G***. Let ***Y =*** {***y***_**1**_, ***y***_**2**_, …, ***y***_|***C***|_} indicate label set, which means there was |***C***| different subcellular location. Our goal is to predict the subcellular location (label) ***y***_***i***_ ***∈ Y*** of a lncRNA (***v***_***i***_ ***∈ V***) by aggregating the node feature(***x***_***i***_) of the lncRNA (***v***_***i***_) and the feature information of its neighbor nodes.

Therefore, the graph consists of three parts, the node-set ***V***, the node features ***X*** and the edge-set ***E***. The construction steps are as follows:

- Step 1: To calculate the cosine similarity in **Step 3**, the low-level features are extracted from ***V =*** {***v***_**1**_, ***v***_**2**_, …, ***v***_***n***_} by k-mer, and then mark them as ***L =*** {***l***_**1**_, ***l***_**2**_, …, ***l***_***n***_};
- Step 2: To learn the high-level features by the classifier, the low-level features extracted from each lncRNA sequence are expressed as the initial features of the corresponding node, forming the node features ***X =*** {***x***_**1**_, ***x***_**2**_, …, ***x***_***n***_};
- Step 3: Calculate the cosine similarity ***S*** between the low-level features ***L*** from **Step 1**. When the cosine similarity ***S***_***i,j***_ between two low-level features ***l***_***i***_ and ***l***_***j***_ is greater than a certain threshold ***τ***, an edge is created for the two nodes(***e***_***i,j***_ ***=*** **1**; otherwise, ***e***_***i,j***_ ***=*** **0**). As shown in eqs. (1) and (2).

1 $${\boldsymbol{S}}_{\boldsymbol{i},\boldsymbol{j}}=\frac{{\boldsymbol{l}}_{\boldsymbol{i}}\bullet {\boldsymbol{l}}_{\boldsymbol{j}}}{\left\Vert {\boldsymbol{l}}_{\boldsymbol{i}}\right\Vert \left\Vert {\boldsymbol{l}}_{\boldsymbol{j}}\right\Vert }$$

2 $${\boldsymbol{e}}_{\boldsymbol{i},\boldsymbol{j}}=\left\{\begin{array}{c}\textbf{1},{\boldsymbol{S}}_{\boldsymbol{i},\boldsymbol{j}}\ge \boldsymbol{\tau} \\ {}\textbf{0},{\boldsymbol{S}}_{\boldsymbol{i},\boldsymbol{j}}<\boldsymbol{\tau} \end{array}\right.$$

**Fig. 3:**
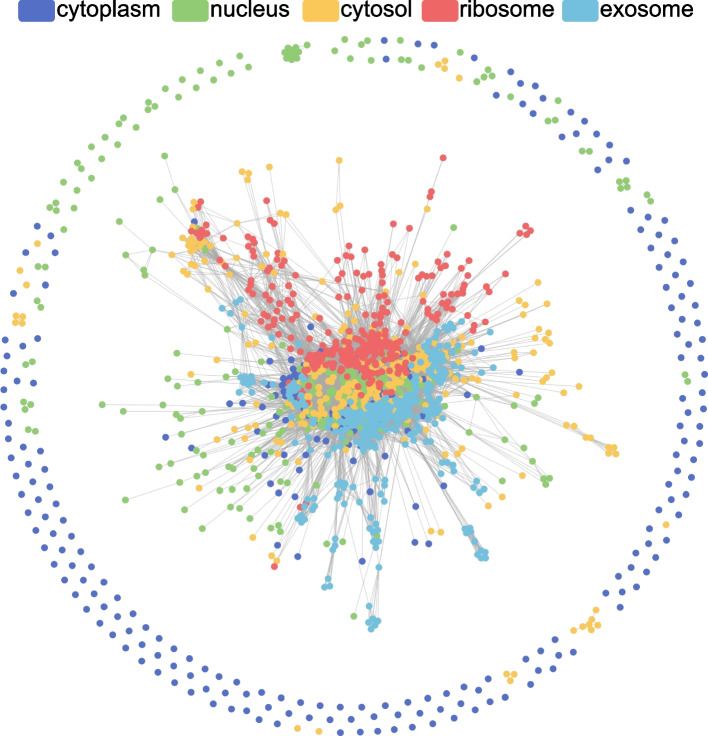
Visualization of similarity graph (the peripheral nodes are isolated nodes)

***τ*** is a hyperparameter, which we will discuss further in Section 3.2. It should be noted that different methods can be used to extract low-level features from lncRNA sequences in Step 1 and Step 2. By experiment comparisons, we found that GM-lncLoc performs best when k-mer was used for both similarity features and node features, as shown in Table 2. In addition, the final constructed graph is allowed to have isolated nodes, which implies support for new lncRNA prediction, as shown in Fig. 3.

**Table 2:** The performance with different features

features of calculating cosine similarity	features of node features	F1	Recall	Acc
**k-mer**(k = 5)	**k-mer**(k = 5)	**0.833**	**0.835**	**0.822**
	**RevKmer**(k = 5)	0.713	0.714	0.721
	**PseDNC**(λ = 150 and ω = 0.3)	0.529	0.530	0.529
**k-mer**(k = 5)	**k-mer**(k = 5)	**0.833**	**0.835**	**0.822**
**RevKmer**(k = 5)		0.743	0.742	0.754
**PseDNC**(λ = 150 and ω = 0.3)		0.662	0.658	0.661

### GNN based on Meta-learning

#### Graph Convolutional Network (GCN)

GCN [36] is a semi-supervised learning graph neural network that can be applied to tasks such as node classification and link prediction. The input of GCN consists of two parts: ***X***_***n × s***_ and ***A***_***n × n***_; where ***X***_***n × s***_ represents the n × s feature matrix, while ***A***_***n × n***_ represents the n × n adjacency matrix. The output matrix is ***Y***_***n ×*** |***C***|_, where |***C***| represents the number of labels, and ***Y***_***i,j***_ represents the probability that node ***v***_***i***_ is predicted to be in the j-th label. The formula of GCN is defined as eq (3).

3 $$\boldsymbol{Y}=\boldsymbol{f}\left(\boldsymbol{X},\boldsymbol{A}\right)=\boldsymbol{\sigma} \left(\boldsymbol{D}{\prime}^{-\frac{\textbf{1}}{\textbf{2}}}\ {\boldsymbol{A}}^{\prime }\ \boldsymbol{D}{\prime}^{-\frac{\textbf{1}}{\textbf{2}}}\ \boldsymbol{XW}\right)$$

where ***A***^’^ ***= A + E***, ***E*** notes an identity matrix; ***D***^′^ is the ***degree matrix***^3^ of ***A***^′^; ***W*** is the weight matrix and ***σ*** notes an activation function.

#### MAML

MAML [33] is an outstanding model in meta-learning because of its simplicity and universality. Meta-learning focuses on learning meta-knowledge from a series of tasks, so as to learn the parameters of new tasks quickly. In MAML, the set of functions {***g***_**1**_, ***g***_**2**_, …, ***g***_***k***_} in Meta-train learn the meta-parameters ***θ***^′^ (meta-knowledge) through ***k*** tasks {***T***_**1**_, ***T***_**2**_, …, ***T***_***k***_}, and then let meta-parameters be used as the initial parameters of the function ***g*** in Meta-test to quickly adapt to the new task *T*. MAML can be simply understood as a training mode: *Pre-training*^4^ from Meta-train + Fine-tuning in Meta-test. This training mode can not only effectively deal with the problem of few-shot learning, but also significantly reduce the training time for new tasks employing meta-parameters, which can be verified by the experiment in **Section 3.5**.

#### GCN based on MAML

**Fig. 4:**
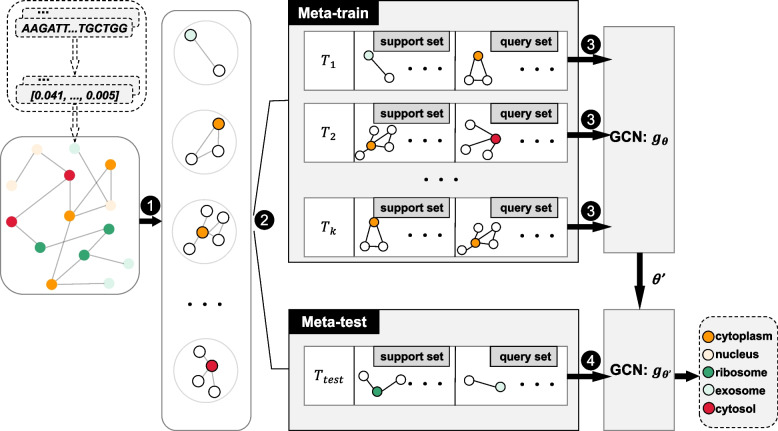
The algorithm flow chart of GCN based on MAML: (1) extracting local graphs according to the neighbor nodes; (2) dividing the local graphs into three datasets (training, validation and testing) and constructing tasks for each dataset; (3) feeding a batch of k support sets into GCN to get k θ and calculating the θ’ based on k query sets and θ’; (4) using the support set of the testing set to fine-tune the GCN with meta-parameter θ’ as the initial parameter and the query set to evaluate the model’s performance. In addition, the validation set is also used to adjust the hyperparameters in this step

The data of lncRNA were transformed into graphical data, while the problem of fewer lncRNA samples still exists. Therefore, we combined GCN and MAML in predicting lncRNA subcellular localization, that is, the training mode of MAML is applied to the training of GCN model. Since the training of MAML is task-based, and tasks need to be constructed by repeatedly sampling from the dataset. To fit the training mode of MAML, local graphs of each node in graph need to be extracted first. The algorithm flow chart is shown in Fig. 4. The details are as follows:

1. Extracting local graph: In **Section 2.2**, we have constructed graph ***G =*** (***V***, ***E***, ***X***) for lncRNA. Then we extract each node {***v***_**1**_, ***v***_**2**_, …, ***v***_***n***_} and its neighbor nodes in graph ***G*** to form the corresponding local graph {***G***_**1**_, ***G***_**2**_, …, ***G***_***n***_} of ***n*** nodes, where ***G***_***i***_ ***∈ G*** represents the local graph of the i-th node, and ***G***_***i***_ ***=*** {***V***_***i***_, ***E***_***i***_, ***X***_***i***_}, ***V***_***i***_ ***=*** {***v***_***i***_} ***∪*** {***v***_***j***_ ***∈ V***|***e***_***i,j***_ ***=*** **1**}, ***E***_***i***_ ***=*** {***e***_***i,j***_ ***∈ E***|***e***_***i,j***_ ***=*** **1**}, ***X***_***i***_ ***=*** {***x***_***i***_} ***∪*** {***x***_***j***_ ***∈ X***|***e***_***i,j***_ ***=*** **1**}. Thus, 1460/1668 local graphs (samples) of lncRNA can be obtained, that is ***D =*** {***G***_**1**_, ***G***_**2**_, …, ***G***_**1460**/**1668**_};
2. Dividing dataset: Firstly, the dataset ***D*** = {***G***_**1**_, ***G***_**2**_, …, ***G***_***n***_} is divided into three data sets: ***D***_***train***_ = {***G***_***a***_, …, ***G***_***o***_}, ***D***_***val***_ = {***G***_***b***_, …, ***G***_***p***_} and ***D***_***test***_ = {***G***_***c***_, …, ***G***_***q***_}, and the following condition are satisfied$$\left\{\begin{array}{c}{\boldsymbol{D}}_{\boldsymbol{train}}\cap {\boldsymbol{D}}_{\boldsymbol{val}}\cap {\boldsymbol{D}}_{\boldsymbol{test}}=\varnothing \\ {}{\boldsymbol{D}}_{\boldsymbol{train}}\cup {\boldsymbol{D}}_{\boldsymbol{val}}\cup {\boldsymbol{D}}_{\boldsymbol{test}}=\boldsymbol{D}\end{array}\right.$$; Then, according to the MAML method, *m* tasks ***T***_***train***_ = {***T***_**1**_, ***T***_**2**_, …, ***T***_***m***_} are composed of randomly selected |***C***| × (***k***_***support***_ + ***k***_***query***_) samples ***G***_***i***_ repeatedly, where |***C***| represents the number of location labels, ***k***_***support***_, ***k***_***query***_ and *m* are the hyperparameter; The samples ***G***_***i***_ in ***D***_***val***_ and ***D***_***test***_ constitute a single task ***T***_***val***_ and ***T***_***test***_ respectively; Finally, each task is further divided into support set and query set, denoted as ***T***_***i*** − ***support***_ and ***T***_***i*** − ***query***_, (respectively;)
3. Meta-train: Firstly, m tasks’ ***T***_***train − support***_ of ***T***_***train***_ are input into m GCNs(i.e. ***f***_***θ***_) with initial parameters ***θ*** for training, and m corresponding parameters {***θ***_**1**_, ***θ***_**2**_, …, ***θ***_***m***_} are obtained after updating respectively; Then, the total loss is calculated for updating ***θ*** by m tasks’ ***T***_***train − query***_ and $\left\{{\boldsymbol{f}}_{{\boldsymbol{\theta}}_{\textbf{1}}},{\boldsymbol{f}}_{{\boldsymbol{\theta}}_{\textbf{2}}},\dots, {\boldsymbol{f}}_{{\boldsymbol{\theta}}_{\boldsymbol{m}}}\right\}$ in ***T***_***train***_. Finally, the optimized meta-parameter ***θ***^’^ is obtained;
4. Meta-test: ***T***_***test − support***_ of ***T***_***test***_ is used to fine-tune the GCN(i.e.***f***_***θ***′_) with meta-parameter ***θ***^’^ as the initial parameter, then ***T***_***test − query***_ is used to evaluate the performance of ***f***_***θ***′_. In the actual training, ***T***_***val***_ is used before the Meta-test in step 4 to verify the model, and then adjust the hyperparameters. Moreover, another graph is constructed by the independent test set, dataset3. Therefore, there are no overlaps between the training data and the independent test dataset.

### Performance evaluation

To evaluate the performance of GM-lncLoc, the following evaluations criterion is performed based on 10-fold cross-validation. In addition to the typical Accuracy (Acc), Recall(R) and F_1_ Score (F1) are also included. The formula is shown below.

4 $$\boldsymbol{Acc}=\frac{\boldsymbol{TP}+\boldsymbol{TN}}{\boldsymbol{TP}+\boldsymbol{TN}+\boldsymbol{FP}+\boldsymbol{FN}}$$

5 $${\boldsymbol{P}}^{\left(\boldsymbol{i}\right)}=\frac{{\boldsymbol{TP}}^{\left(\boldsymbol{i}\right)}}{{\boldsymbol{TP}}^{\left(\boldsymbol{i}\right)}+{\boldsymbol{FP}}^{\left(\boldsymbol{i}\right)}}$$

6 $${\boldsymbol{R}}^{\left(\boldsymbol{i}\right)}=\frac{{\boldsymbol{TP}}^{\left(\boldsymbol{i}\right)}}{{\boldsymbol{TP}}^{\left(\boldsymbol{i}\right)}+{\boldsymbol{FN}}^{\left(\boldsymbol{i}\right)}}$$

7 $$\boldsymbol{F}\textbf{1}=\frac{\textbf{1}}{\left|\boldsymbol{C}\right|}\sum_{\boldsymbol{i}=\textbf{1}}^{\left|\boldsymbol{C}\right|}\textbf{2}\times \frac{{\boldsymbol{P}}^{\left(\boldsymbol{i}\right)}\times {\boldsymbol{R}}^{\left(\boldsymbol{i}\right)}}{{\boldsymbol{P}}^{\left(\boldsymbol{i}\right)}+{\boldsymbol{R}}^{\left(\boldsymbol{i}\right)}}$$

8 $$\boldsymbol{R}=\frac{\textbf{1}}{\left|\boldsymbol{C}\right|}\sum_{\boldsymbol{i}=\textbf{1}}^{\left|\boldsymbol{C}\right|}{\boldsymbol{R}}^{\left(\boldsymbol{i}\right)}$$

where ***TP***, ***FP*** and ***FN*** represent true positive, false positive and false negative, respectively. ***P*** represents Precision, and |***C***| represents the number of location labels.

## Results and discussion

### Performance comparison of different node features

To explore the effect of feature extraction method, we compared the prediction results of three low-level feature extraction methods, including k-mer [39], RevKmer [40, 41] and PseDNC [42–44], which is on the basis of the previous study [14, 25–31] in dataset1. As shown in Table 2, the k values of both k-mer and RevKmer are 5, and the **λ** and **ω** of PseDNC are set to 150 and 0.3 respectively. First of all, the low-level features extracted by k-mer are fixed as the features of calculating cosine similarity, and comparing the features extracted by the three methods as node features, then the accuracy of 82.2, 72.1, and 52.9% are obtained, respectively. Next, fixing the low-level features extracted by k-mer as node features and comparing the low-level features extracted by the three methods as the features of calculating cosine similarity, the accuracy of 82.2, 75.4, and 66.1%, are obtained, respectively.

As we can see from the results, GM-lncLoc shows the best performance when the low-level features extracted by k-mer are used in calculating cosine similarity and the node features. RevKmer removes some frequency of base sequences on the basis of k-mer, which is essentially a dimensionality reduction operation and may lose some information; PseDNC is a method based on pseudo dinucleotide composition, which may be limited to dinucleotide and fail to extract more critical information.

### Performance comparison of different values of threshold τ

When constructing the graph, the value of threshold ***τ*** directly determines the structure of the graph, especially the number of edges. Therefore, it is necessary to discuss the influence of threshold ***τ*** on GM-lncLoc. While the value of ***τ*** is set from 0.4 to 0.9, we compared the performance of GM-lncLoc in dataset1. In addition, to evaluate the impact of edges on the model, we also tallied the number of isolated nodes and edges in the graph, and the proportion of key edges.^5^

As shown in Table 3, as the value of ***τ*** increases, the number of isolated nodes in the graph also increases, while the number of edges decreases. The number of isolated nodes and edges is directly related to the structural information of the graph. Meanwhile, the proportion of key edges and the overall performance of GM-lncLoc are improving as the value of ***τ*** increases from 0.4 to 0.7. However, when ***τ*** rises from 0.7 to 0.9, although the proportion of key edges also rises from 91.3 to 99.4%, the performance of GM-lncLoc deteriorates. It indicates that the performance of GM-lncLoc is not only related to the number of isolated nodes and edges but also related to the proportion of the key edge.

**Table 3:** The performance with different threshold τ

τ	isolated nodes	edges	key edges(%)	F1	Recall	Acc
0.4	251	90,210	44.7	0.895_±0.012_	0.895_±0.012_	0.897_±0.013_
0.5	324	31,478	73.9	0.922_±0.017_	0.923_±0.011_	0.924_±0.012_
0.6	352	19,942	84.8	0.928_±0.013_	0.927_±0.013_	0.929_±0.014_
0.7	367	13,872	91.3	**0.933**_**±0.011**_	**0.933**_**±0.013**_	**0.934**_**±0.01**_
0.8	408	9072	96.4	0.915_±0.015_	0.914_±0.014_	0.914_±0.012_
0.9	525	4692	99.4	0.843_±0.011_	0.834_±0.014_	0.834_±0.012_

### Performance comparison of different k values in k-mer

**Fig. 5:**
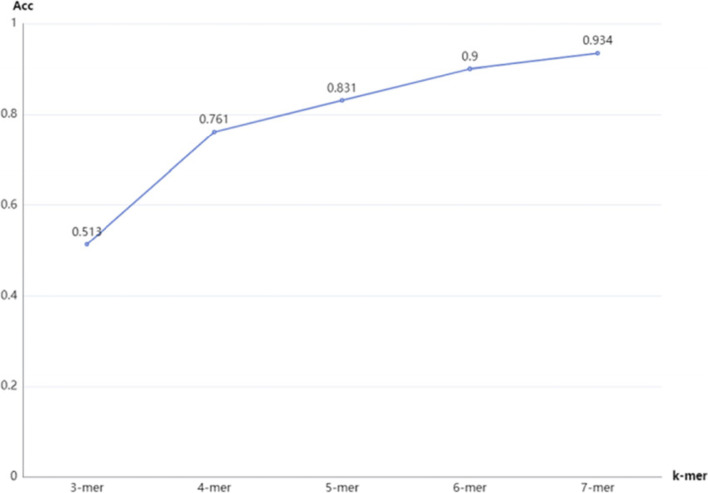
The accuracy with different k values in k-mer

To explore the effect of k value on our model, the k value is set from 3 to 7. The comparative experiment in dataset1 is shown in Table 4**.** and Fig. 5. Since the dimension of the 7-mer frequency vector is 16,384, the higher k-mer frequency feature is not conducted in our experiments considering the time cost and equipment conditions. It can be apparently seen from Table 4**.** that the performance of GM-lncLoc improves as the increase of k value.

**Table 4:** The performance with different k values in k-mer

	F1	Recall	Acc	Time(s)
3-mer	0.558_±0.016_	0.560_±0.011_	0.513_±0.014_	5246
4-mer	0.760_±0.013_	0.761_±0.013_	0.761_±0.014_	6250
5-mer	0.829_±0.014_	0.830_±0.013_	0.831_±0.015_	8378
6-mer	0.897_±0.013_	0.898_±0.014_	0.900_±0.011_	23,890
7-mer	**0.933**_**±0.011**_	**0.933**_**±0.013**_	**0.934**_**±0.01**_	48,993

**Fig. 6:**
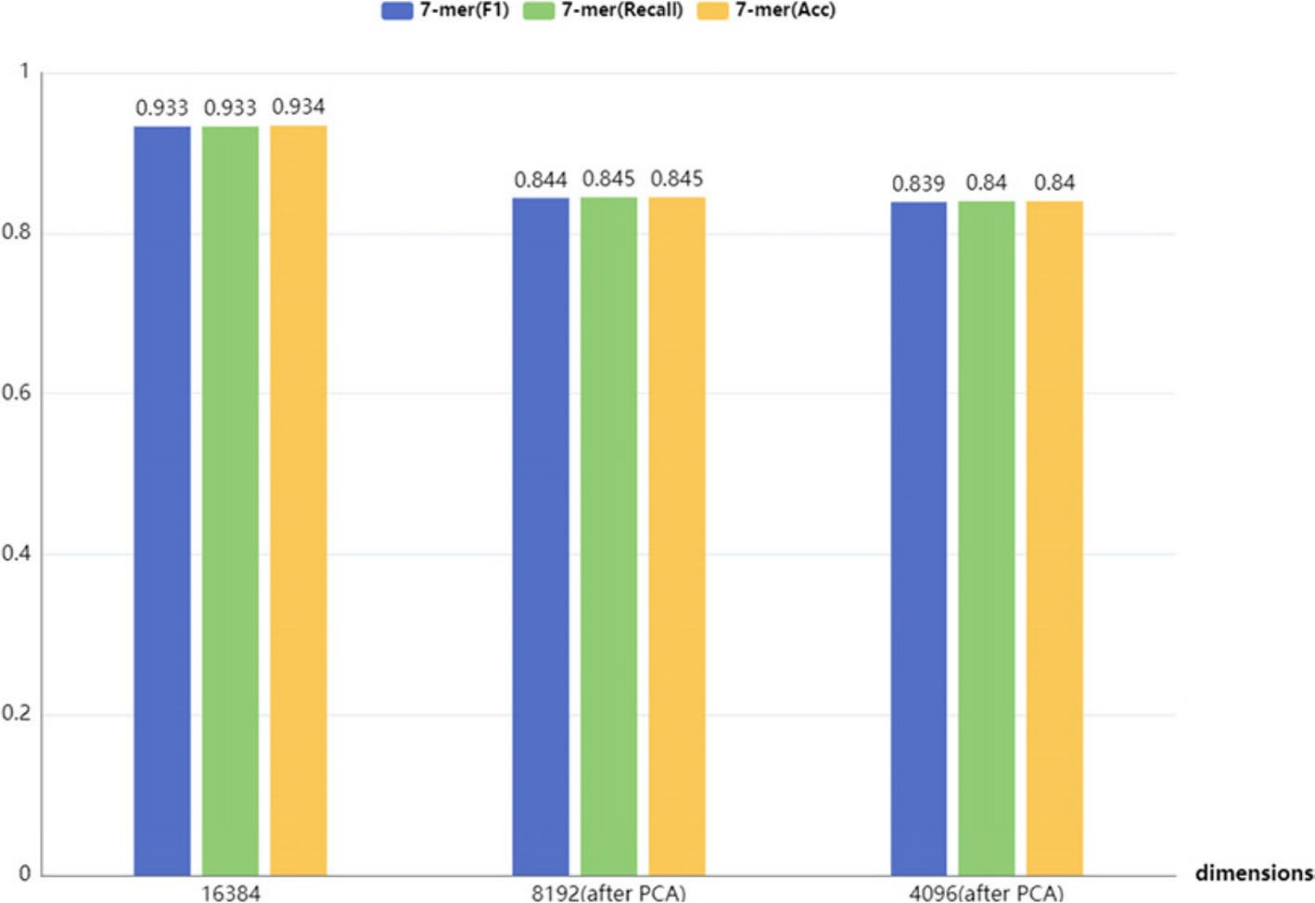
The performance after dimension reduction of features from 7-mer

In addition, the dimension may be too high when the feature is the 7-mer frequency vector. Therefore, we also tried to utilize the PCA algorithm to reduce the dimension of the node features from 16,384 to 8192 and 4096, and then compared their performance. It can be seen in Fig. 6 that the accuracy after dimension reduction has not been improved, but rather decreased. We believe that the loss of some information after the dimensionality reduction operation is what makes it ineffective.

### Performance comparison of different number of neighbor node’s layer

**Fig. 7:**
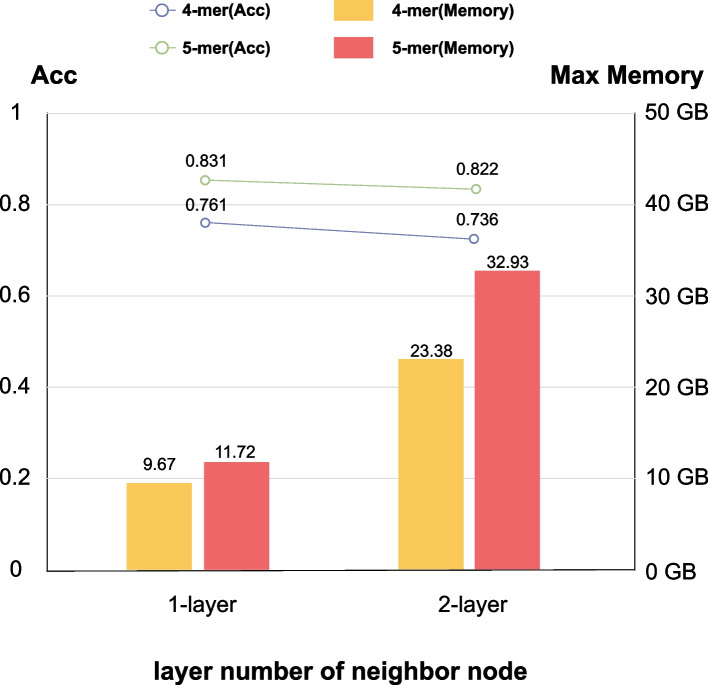
The performance with different number of neighbor node’s layer

GCN aggregates information about neighbor nodes during computing node embedding. In this paper, if there is an edge between node A and node B, node B is called one of the first-layer neighbor nodes of node A. Furthermore, if there is also an edge between node B and node C, node C is called one of the second-layer neighbor nodes of node A. Thus, when the neighbor node information is aggregated, there is a difference between the neighbor node information of the first layer and that of the first two layers. We conducted a relevant experimental comparison in dataset1, and the results are shown in Fig. 7. It can be seen that the model performance on the first-layer neighbor aggregation is slightly higher than that of the first two layers. However, the latter consumes 2 to 3 times as much memory than the former in terms of memory consumption. As a result, the first-layer neighbor aggregation is adopted in our experiments.

Among them, memory consumption is in line with our intuitive understanding. The number of neighbor nodes at the first two layers must be larger than that of the neighbor nodes at the first layer, so more memory is required. In addition, the accuracy of the neighbor node information of the first layer of aggregation is higher than that of the neighbor node information of the first two layers of aggregation, which is also consistent with the theoretical proof in Kexin Huang [35] et al., which proves that the interaction between two nodes decreases exponentially as their distance increases. In other word, as the distance increases, the number of neighbor nodes increases exponentially, while the information provided by neighbor nodes decreases exponentially. Therefore, the further the distance, the less efficient the information aggregation is.

### Performance comparison of GM-lncLoc and GCN

To validate the effectiveness of MAML, we trained GCN alone in dataset1. As shown in Table 5, the results of GCN alone for predicting lncRNA subcellular localization are not good due to the limited amount of data. On the contrary, GM-lncLoc is able to predict lncRNA subcellular localization more effectively with about 0.4 higher accuracy than GCN alone. In the experiment process, we also find that it only takes about 34.4 seconds to complete the training using meta-parameters as the initial parameters of the meta-test task, while it takes about 325.3 seconds for GCN to complete the training. From the perspective of training duration, GCN took nearly 9.5 times longer than training with meta-parameters, which indicates that the meta-parameters obtained by GM-lncLoc can significantly improve the training efficiency.

**Table 5:** The performance comparison of GCN and GM-lncLoc (Ours)

	F1	Recall	Acc
GCN	–	–	0.531
**GM-lncLoc (Ours)**	**0.933**	**0.933**	**0.934**

### Performance comparison with other methods

In this section, we utilize the method of 10-fold cross-validation to compare the GM-lncLoc with previous methods, as shown in Table 6 and Table 7**.**^6^ It is evident that GM-lncLoc has achieved the best results both on the dataset1 and dataset2. In the dataset1, the accuracy of GM-lncLoc is about 34.3% higher than lncLocator [25]; In the dataset2, the accuracy of GM-lncLoc is about 1.8% higher than the current highest LncLocPred [30]. It demonstrates the superiority of our proposed GM-lncLoc in lncRNA subcellular localization prediction. In particular, the samples are more imbalanced in the dataset1, and our method provides a significant improvement over existing methods. This shows that our method is more advantageous in the case of an imbalanced sample. To improve the persuasion, we set all the samples in the test set as real samples in dataset1 and dataset2 and obtained an accuracy of 90.3 and 93.1%, respectively. Although the accuracy is slightly lower than that of the 10-fold cross-validation method, it is still better than other methods. Moreover, we compare GM-lncLoc with the three methods, iLoc-lncRNA, Locate-R and LncLocPred, in the independent test set (dataset3), and GM-lncLoc attains a better accuracy, 46.21%. Besides, F1 and Recall are 0.469 and 0.463, respectively. However, LncLocPred [25] had not provided other performance evaluations in iLoc-lncRNA, Locate-R and LncLocPred. As shown in Table 8, the result indicates that our model does not depend on a particular dataset, which is better in generalization. To provide strong support to the research, we describe the algorithms and features used for each method in Table 9.

**Table 6:** Comparison with existing state-of-the-art methods (dataset1 with 5 subcellular compartments)

Method	F1	Recall	Acc(%)
lncLocator [25]	0.367	0.363	59.1
DeepLncLoc [31]	0.563	0.524	53.7
**GM-lncLoc (Ours)**	0.933	0.933	93.4
**GM-lncLoc** (the test set consists of real samples)	0.901	0.902	90.3

**Table 7:** Comparison with existing state-of-the-art methods (dataset2 with 4 subcellular compartments)

Method	Location	Sensitivity(%)	Specificity(%)	MCC	Acc(%)
iLoc-lncRNA [14]	Cytoplasm	99.06	67.68	0.742	86.72
	Nucleus	77.56	97.59	0.796	
	Ribosome	46.51	99.83	0.652	
	Exosome	16.67	1.00	0.400	
Locate-R [28]	Cytoplasm	84.74	89.10	0.725	90.69
	Nucleus	65.92	95.15	0.658	
	Ribosome	100.00	98.37	0.970	
	Exosome	100.00	99.17	0.978	
LncLocPred [30]	Cytoplasm	99.10	85.60	0.876	92.37
	Nucleus	96.80	96.80	0.915	
	Ribosome	60.50	99.80	0.751	
	Exosome	20.00	100.00	0.439	
Proposed by Yang et al. [29]	Cytoplasm	100.00	84.14	0.880	90.37
	Nucleus	82.47	97.14	0.821	
	Ribosome	41.86	99.83	0.615	
	Exosome	66.67	98.21	0.639	
**GM-lncLoc (Ours)**	Cytoplasm	93.21	96.06	0.879	94.20
	Nucleus	88.85	98.21	0.889	
	Ribosome	96.80	98.99	0.959	
	Exosome	99.07	99.38	0.982	
**GM-lncLoc** (the test set consists of real samples)	Cytoplasm	99.00	92.37	0.860	93.00
	Nucleus	73.80	99.47	0.811	
	Ribosome	99.20	99.83	0.991	
	Exosome	100.00	99.00	0.980

**Table 8:** Comparison with other methods on the independent dataset (dataset3)

Method	Acc(%)
iLoc-lncRNA [14]	35.86
Locate-R [28]	38.64
LncLocPred [30]	44.44
**GM-lncLoc (Ours)**	46.21

**Table 9:** Description of the algorithms and features used for each method

Method	Feature	Oversampling	Algorithm	The number of subcellular compartments
DeepLncRNA [26]	k-mer, Genome loci, RNA binding motifs	–	Neural networks	2
lncLocator 2.0 [27]	–	–	CNN, LSTM, Multi-layer perceptron	2
iLoc-lncRNA [14]	PseKNC	–	SVM	4
Locate-R [28]	k-mer, n-gapped k-mer	SMOTE	Locally Deep SVM	4
LncLocPred [30]	k-mer, PseDNC, Triplet	–	Logistic regression	4
lncLocator [25]	k-mer	SOS [55]	Random forest, SVM, Neural networks	5
DeepLncLoc [31]	k-mer	–	TextCNN	5
GM-lncLoc **(Ours)**	k-mer	SMOTE	GCN based on MAML	4 or 5

On the one hand, GM-lncLoc is based on GNN and is able to extract high-level features from low-level features of lncRNA sequences to complete classification tasks, while traditional machine learning methods complete classification based on low-level features of lncRNA sequences; On the other hand, GM-lncLoc extract correlation information between lncRNAs based on sequence information, which is unable to be achieved by previous methods. The comparison experiments between GM-lncLoc and previous methods, especially lncLocator, demonstrate the significance of graph structure information for GM-lncLoc. lncLocator is also based only on the low-level features k-mer and utilizes the over-sampling method to augment the dataset. However, our GM-lncLoc obtains 93.4% accuracy based on the graph, while the accuracy of lncLocator only achieves 59.1%. In addition, our model also utilizes a few-shot training model, so that better results can be obtained in lncRNA subcellular localization problems with a limited number of samples.

## Conclusion

In conclusion, our proposed GM-lncLoc based on the combination of Graph Neural Network and Meta-learning is a totally new method for lncRNA subcellular localization prediction. On the one hand, a graph is constructed for the initial data, which is not used in the previous approach; On the other hand, Graph Neural Network and Meta-learning are modeled jointly, which is able to effectively predict lncRNA subcellular localization with only a small number of samples, and obtain the meta-parameters for quickly learning of new lncRNA subcellular localization tasks. The experimental results from lncRNA subcellular localization prediction demonstrate that GM-lncLoc is effective and promising. Even more important, the advantages of GM-lncLoc will become more evident with the addition of new data in the lncRNA database due to the generalization ability of meta-parameters. We have reason to believe that GM-lncLoc can greatly contribute to the further study of lncRNA functional mechanisms in biology.

## Data Availability

The datasets and source code are available at https://github.com/JunzheCai/GM-lncLoc.

## Footnotes

1
http://www.csbio.sjtu.edu.cn/bioinf/lncLocator/
http://lin-group.cn/server/iLoc-LncRNA/download.php

2
https://github.com/jademyC1221/lncLocPred/tree/master/lncLocPred/supplementary%20material

3 $$D{\prime}_{i,j}=\left\{\begin{array}{c}\mathit{\deg}\left({v}_i\right), if\ i=j\\ {}0, othewise\end{array}\right.$$, where *deg(v*_*i*_*)* notes the degree of the vertex *v*_*i*_.

4 Pre-training usually requires a large amount of data, but MAML is originally proposed for few-shot learning, which samples the same sample multiple times when generating a series of tasks in Meta-train [33]**.** Here it can be understood as a special kind of pre-training.

5 For the convenience of expression, we call the edge connecting two nodes of the same class the key edge.

6 In order to facilitate comparison with the methods with dataset2, we introduce other evaluations in Table 7, including Sensitivity, Specificity and MCC.
